# The predictive ability of routinely collected laboratory markers for surgically treated spinal metastases: a retrospective single institution study

**DOI:** 10.1186/s12885-022-10334-8

**Published:** 2022-11-29

**Authors:** Zhehuang Li, Lingling Huang, Bairu Guo, Peng Zhang, Jiaqiang Wang, Xin Wang, Weitao Yao

**Affiliations:** Department of Musculoskeletal Oncology, Affiliated Cancer Hospital of Zhengzhou University, Henan Cancer Hospital, Zhengzhou, 45000 Henan China

**Keywords:** Spine metastases, prognosis, nomogram, survival, laboratory markers

## Abstract

**Purpose:**

We aimed to identify effective routinely collected laboratory biomarkers for predicting postoperative outcomes in surgically treated spinal metastases and attempted to establish an effective prediction model.

**Methods:**

This study included 268 patients with spinal metastases surgically treated at a single institution. We evaluated patient laboratory biomarkers to determine trends to predict survival. The markers included white blood cell (WBC) count, platelet count, neutrophil count, lymphocyte count, hemoglobin, albumin, alkaline phosphatase, creatinine, total bilirubin, calcium, international normalized ratio (INR), platelet-to-lymphocyte ratio (PLR), and neutrophil-to-lymphocyte ratio (NLR). A nomogram based on laboratory markers was established to predict postoperative 90-day and 1-year survival. The discrimination and calibration were validated using concordance index (C-index), area under curves (AUC) from receiver operating characteristic curves, and calibration curves. Another 47 patients were used as a validation group to test the accuracy of the nomogram. The prediction accuracy of the nomogram was compared to Tomita, revised Tokuhashi, modified Bauer, and Skeletal Oncology Research Group machine-learning (SORG ML).

**Results:**

WBC, lymphocyte count, albumin, and creatinine were shown to be the independent prognostic factors. The four predictive laboratory markers and primary tumor, were incorporated into the nomogram to predict the 90-day and 1-year survival probability. The nomogram performed good with a C-index of 0.706 (0.702–0.710). For predicting 90-day survival, the AUC in the training group and the validation group was 0.740 (0.660–0.819) and 0.795 (0.568–1.000), respectively. For predicting 1-year survival, the AUC in the training group and the validation group was 0.765 (0.709–0.822) and 0.712 (0.547–0.877), respectively. Our nomogram seems to have better predictive accuracy than Tomita, revised Tokuhashi, and modified Bauer, alongside comparable prediction ability to SORG ML.

**Conclusions:**

Our study confirmed that routinely collected laboratory markers are closely associated with the prognosis of spinal metastases. A nomogram based on primary tumor, WBC, lymphocyte count, albumin, and creatinine, could accurately predict postoperative survival for patients with spinal metastases.

## Introduction

Spinal metastasis occurs in approximately 10% of all patients with cancer and up to 40% of patients with metastatic disease [1, 2]. Although surgery can improve quality of life for indicated patients [3–5], the decision to undergo surgery should be balanced against the limited life expectancy and high disease burden in many of these patients.

Accurate assessment of expected survival is an important prerequisite for an optimized therapeutic plan and objective physician-patient communication [6–9]. The accuracy of prediction algorithms can be improved by adding useful variables [10–13]. The predictive value of laboratory parameters for a variety of tumors have been widely recognized [14–17]; for spinal metastases, current evidence suggests serum albumin, hemoglobin, white blood cell (WBC) count, platelet-lymphocyte ratio (PLR), neutrophil to lymphocyte ratio (NLR), alkaline phosphatase, et al. (Table 1) [13]. Laboratory parameters can be measured and objectively evaluated to identify normal biological or pathogenic processes including the nutritional status, the reserve function of the organ systems, and the inflammatory status of the body [11, 18, 22, 23]. Compared to commonly accepted prognostic factors, the prediction ability of routinely collected laboratory markers has received increasing attention in recent years. However, the prognostic markers suggested in different studies vary. No previous studies have comprehensively assessed the potential laboratory markers.

**Table 1:** Scoring systems that incorporating laboratory markers

Scoring system	Authors & Year	Prognostic Laboratory Markers	Other predictor in the scoring system
Revised Katagiri	Katagiri et al. 2014 [18]	Abnormal: CRP ≥0.4 mg/ dL, LDH ≥250 IU/L, albumin < 3.7 g/dL Critical: platelets < 100,000/μL, Ca2+ ≥10.3 mg/dL, or total bilirubin ≥1.4 mg/dL	Eastern Cooperative Oncology Group (ECOG) grade, primary tumor type, presence of visceral or brain metastases, prior chemotherapy, presence of multiple skeletal fractures
The New England Spinal Metastasis Score (NESMS)	Ghori et al. 2015 [19]	Albumin: ≤3.4 g/dL vs ≥ 3.5 g/dL	Modified Bauer score, ambulatory status
SORG classic	Paulino Pereira et al. 2016 [20]	WBC: ≥11 × 10^3^/μL vs < 11 × 10^3^/μL Hgb: ≤10 g/dL	Age, ECOG grade, primary pathology, presence of multiple spine metastases, presence of visceral or brain metastases, prior systemic therapy
SORG nomogram	Paulino Pereira et al. 2016 [20]	Hgb: from 6 to 18 g/dL WBC: from 2 to 34 × 10^3^/μL	Age, prior systemic therapy, visceral/brain metastases, mobile spine metastases, primary tumor type, ECOG grade
SORG ML	Karhade et al. 2019 [11]	Hgb: from 7 to 17 g/dL Platelet: from 20 to 900 × 10^3^/μL Absolute Lymphocyte: from 0.06 to 4.5 × 10^3^/μL Absolute Neutrophil: from 0.8 to 60 × 10^3^/μL Creatinine: from 0.3 to 6 mg/dL INR: from 0.9 to 2.1 WBC: from 2 to 50 × 10^3^/μL Alkaline Phosphatase: from 30 to 1200 IU/L Albumin: from 2.1 to 5.2 g/dL	Primary tumor histology, ECOG, ASIA, CCI, visceral metastases, brain metastases, prior systemic therapy, no. of spine metastases, BMI
Scoring rubric using PLR and serum albumin	Schoenfeld et al. 2019 [21]	PLR: ≤180 vs > 180 Albumin: ≤3.5 g/dL vs > 3.5 g/dL	None

In this study, we aimed to identify effective biomarkers for predicting postoperative outcomes in surgically treated spinal metastases and attempted to establish an effective prognosis prediction model.

## Methods

### Study design and subject selection

We retrospectively reviewed data from all patients who underwent surgery for spinal metastases at our institution from January 2017–August 2020. These patients were used to develop the prediction model. Another group of patients undergoing surgery from September 2020 to January 2021 was used as the validation group. Our institutional review board approved a waiver due to its retrospective nature. A multidisciplinary team managed the therapeutic approaches. The decision to perform surgery was based on the patient’s medical fitness, clinical presentation (neurologic deficit, spinal instability, intractable pain), oncological status, and feasibility of surgical treatment. Patient follow-ups were conducted prospectively by the Linkdoc Company under the authority of the hospital. Postoperative follow-up evaluations were scheduled 3, 6, and 12 months after the first year, every 6 months for the next 2 years, and annually thereafter.

The inclusion criteria were: (1) The patient was > 18 years when surgery was performed; (2) the diagnosis of spinal metastasis was pathologically confirmed; (3) the entire data needed for survival assessment using the scoring systems was available in the electronic medical record. The exclusion criteria were: (1) Patients lost to follow-up within 1 year after surgery without definitive determination of survival; (2) the surgical procedure was percutaneous vertebroplasty alone.

### Predictors

Primary tumor histology and laboratory markers were used to construct the prediction model. Primary tumor histology was classified into slow growth group, moderate growth group, and rapid growth group according to the study of Katagiri et al. [11, 18].

Baseline laboratory marker values were defined as results within 2 weeks prior to surgery; if a laboratory item was repeated within this period, the result closest to the time of surgery was selected. The laboratory markers analyzed in this study included white blood cell count (WBC,× 10^3^ per microliter [μL]), hemoglobin (grams per deciliter [g/dL]), platelet count (× 10^3^/μL), neutrophil count (× 10^3^/μL), lymphocyte count (× 10^3^/μL), albumin (g/dL), alkaline phosphatase (international units per liter [IU/L]), creatinine (mg/dL), total bilirubin (mg/dL), calcium (milligrams per deciliter [mg/dL]), international normalized ratio (INR), platelet-to-lymphocyte ratio (PLR), and neutrophil-to-lymphocyte ratio (NLR).

### Statistical analysis

To compare the patient and tumor data between the training group and the validation group, the Student t test was used for continuous variables. The chisquare tests (Pearson or Fisher exact test as appropriate) were used for categorical variables.

Log-rank tests were used to perform survival analysis. Kaplan-Meier analysis was used to create the survival curves. We computed univariate Cox analysis for all markers, factors with *P* value ≤0.1 were subjected to multivariate Cox analysis. The optimal cutoff laboratory marker value to create survival curves was determined through a log-rank test by taking the split with the highest significance [24].

The multivariate analysis guided the development of a nomogram. Each β regression coefficient of every parameter was proportionally transformed to a scale from 0 to 100. The factor with the highest β coefficient was assigned 100 points. The total points are mapped to obtain the 90-day, and 1-year survival probability.

Concordance index (C-index) and area under curves (AUC) from receiver operating characteristic (ROC) curves was used to measure discrimination. Calibration curves were used to evaluate calibration. Using AUC for survival at 90-day and 1-year, the nomogram was compared to Tomita, revised Tokuhashi, modified Bauer, and SORG ML.

The data were statistically analyzed using R version 4.1.3 for Windows (R Project for Statistical Computing, http://www.r-project.org/). A two-tailed *P* value of < 0.05 were considered statistically significant.

## Results

### Patients and characteristics

Based on the inclusion and exclusion criteria, 268 patients (124 male, 144 female) with an average age of 55.4 ± 10.6 years (range, 23–78 years) were included to assessed the potential prognostic laboratory markers and to develop a prediction model. Neurological deficits were observed in 41.8% of the patients preoperatively. The primary tumor histology was as follows: lung (*n* = 71, 26.5%), breast (*n* = 52, 19.4%), multiple myeloma (*n* = 21, 7.8%), liver (*n* = 15, 5.6%), and other (*n* = 109, 40.7%). Baseline markers (as mean ± SD) were: WBC, 6.73 ± 3.04 × 103/μL; hemoglobin, 12.3 ± 1.8 g/dL; lymphocyte count, 1.38 ± 0.71 × 103/μL; albumin, 4.0 ± 0.5 g/dL; NLR, 4.9 ± 4.7; and PLR, 216.6 ± 157.8. The baseline patient characteristics of the patients are summarized in Table 2.

**Table 2:** Baseline characteristics of the training group and the validation group

Characteristic	Training ***n*** = 268	Validation ***n*** = 47	***P***
**Demographic**			0.478
Age (years)	55.4 ± 10.6	56.7 ± 11.3	
Male sex	124 (46.3%)	22 (46.8%)	
**Clinical and surgical**			
Preoperative ASIA impairment scale			0.902
A to C	51 (19.0%)	8 (17.0%)	
D or E	217 (81.0%)	39 (83.0%)	
ECOG performance status			0.070
Score 0–2 (≤50% of waking hours bed or chair bound)	103 (38.5%)	11 (23.4%)	
Score 3–4 (> 50% of waking hours bed or chair bound)	165 (61.5%)	36 (76.6%)	
Surgery			0.252
Corpectomy or vertebrectomy with stabilization	117 (43.7%)	24 (51.1%)	
Decompression and stabilization	70 (26.1%)	13 (27.7%)	
Decompression alone	80 (29.9%)	9 (19.1%)	
Stabilization alone	1 (0.4%)	1 (2.1%)	
**Oncologic status**			
No. of spine metastases			0.136
1 level	91 (34.0%)	12 (25.5%)	
2 levels	35 (13.1%)	3 (6.4%)	
≥ 3 levels	142 (53.0%)	32 (68.1%)	
Visceral metastases at time of surgery			0.812
None	148 (55.2%)	28 (59.6%)	
Liver or lung	99 (36.9%)	19 (40.4%)	
Brain	30 (11.2%)	4 (8.5%)	
Prior local radiotherapy	26 (9.7%)	4 (8.5%)	1.000
Previous systemic therapy	147 (54.9%)	26 (55.3%)	1.000
**Laboratory data**			
WBC count (×10^3^/μL)	6.73 ± 3.04	7.06 ± 3.27	0.513
Hemoglobin (g/dL)	12.3 ± 1.8	12.2 ± 2.0	0.899
Platelet count (×10^3^/μL)	226.6 ± 76.8	230.4 ± 114.1	0.828
Neutrophil count (×10^3^/μL)	4.86 ± 2.58	5.03 ± 2.88	0.708
Lymphocyte count (×10^3^/μL)	1.38 ± 0.71	1.47 ± 0.73	0.459
Albumin (g/dL)	4.0 ± 0.5	3.9 ± 0.5	0.644
Alkaline phosphatase (IU/L)	124.0 ± 148.6	103.8 ± 47.3	0.079
Creatinine (mg/dL)	0.71 ± 0.21	0.66 ± 0.27	0.214
Total Bilirubin (mg/dL)	10.5 ± 6.0	12.4 ± 8.3	0.152
Ca2+ (mg/dL)	2.3 ± 0.7	2.3 ± 0.2	0.971
INR	0.97 ± 0.13	0.99 ± 0.09	0.209
NLR	4.9 ± 4.7	4.3 ± 3.3	0.274
PLR	216.6 ± 157.8	200.5 ± 177.9	0.564

*ASIA* American Spinal Injury Association, *BMI* Body mass index, *ECOG* Eastern Cooperative Oncology Group

A total of 147 (54.9%) patients received preoperative systematic therapy. Of these, 124 (46.3%) received chemotherapy, 42 (15.7%) received targeted therapy, 39 (14.6%) received endocrine therapy, and 9 (3.3%) received immunotherapy. Preoperative radiotherapy was performed in 26 (9.7%) patients. The surgeries were all performed under general anesthesia in a single stage. Surgeries lasted an average time of 241.9 ± 101.6 min, with an average blood loss of 1081.0 ± 1008.3 mL. The posterior approach was employed in 241 (89.9%) cases, anterior approach in 20 (7.5%), and combined approach in 7 (2.6%). Corpectomy or vertebrectomy with stabilization was performed in 117 (43.7%) patients, decompression and stabilization in 70 (26.1%), decompression alone in 80 (29.9%), and stabilization alone in one (0.4%).

Another 47 surgically treated patients were also enrolled to validate the accuracy of the prediction model developing using the training group. The datails of the validation group were also summarized in Table 2.

### The univariate and multivariate analysis of the laboratory markers

Based on the univariate Cox analysis, nine laboratory markers were subjected for multivariate Cox analysis: WBC (*P* = 0.003), hemoglobin (*P* < 0.001), lymphocyte count (*P* < 0.001), neutrophil count (*P* < 0.001), albumin (*P* < 0.001), creatinine (*P* = 0.055), INR (*P* = 0.001), NLR (*P* < 0.001), and PLR (*P* = 0.009). The multivariate analysis identified WBC (*P* = 0.004), lymphocyte count (*P* = 0.004), albumin (*P* < 0.001), and creatinine (*P* = 0.048) as independent prognostic factors. The results of the univariate and multivariate analysis are summarized in Table 3.

**Table 3:** Univariate and multivariate analysis of the potential prognostic laboratory markers with primary site

Variables	Univariate analysis		Multivariate analysis	
	HR (95% CI)	*P*	HR (95% CI)	*P*
Primary site				
Slow growth	Ref	Ref		Ref
Moderate growth	2.376 (1.525–3.703)	< 0.001*	1.942 (1.230–3.069)	0.004**
Rapid growth	3.396 (2.278–5.061)	< 0.001*	2.988 (1.975–4.520)	< 0.001**
WBC	1.074 (1.024–1.125)	0.003*	1.285 (1.086–1.520)	0.004**
Hemoglobin	0.820 (0.747–0.900)	< 0.001*	0.992 (0.982–1.001)	0.914
Platelet	1.000 (0.997–1.002)	0.641		
Lymphocyte count	0.663 (0.526–0.835)	< 0.001*	0.511 (0.322–0.809)	0.004**
Neutrophil count	1.098 (1.044–1.154)	< 0.001*	0.835 (0.678–1.029)	0.091
Albumin	0.385 (0.273–0.541)	< 0.001*	0.501 (0.342–0.734)	< 0.001**
Alkaline phosphatase	1.000 (0.999–1.001)	0.825		
Creatinine	2.082 (0.984–4.405)	0.055*	2.126 (1.006–4.491)	0.048**
Total Bilirubin	0.992 (0.962–1.022)	0.581		
Ca2+	1.103 (0.916–1.327)	0.302		
INR	3.662 (1.647–8.143)	0.001*	1.373 (0.482–3.914)	0.553
NLR	1.048 (1.020–1.077)	< 0.001*	0.962 (0.888–1.043)	0.352
PLR	1.001 (1.000–1.002)	0.009*	1.000 (0.999–1.002)	0.650

* *P* value of < 0.1 in univariate analysis and subjected to multivariate analysis

** *P* value of < 0.05 in multivariate analysis and used for nomogram construction

**Fig. 1:**
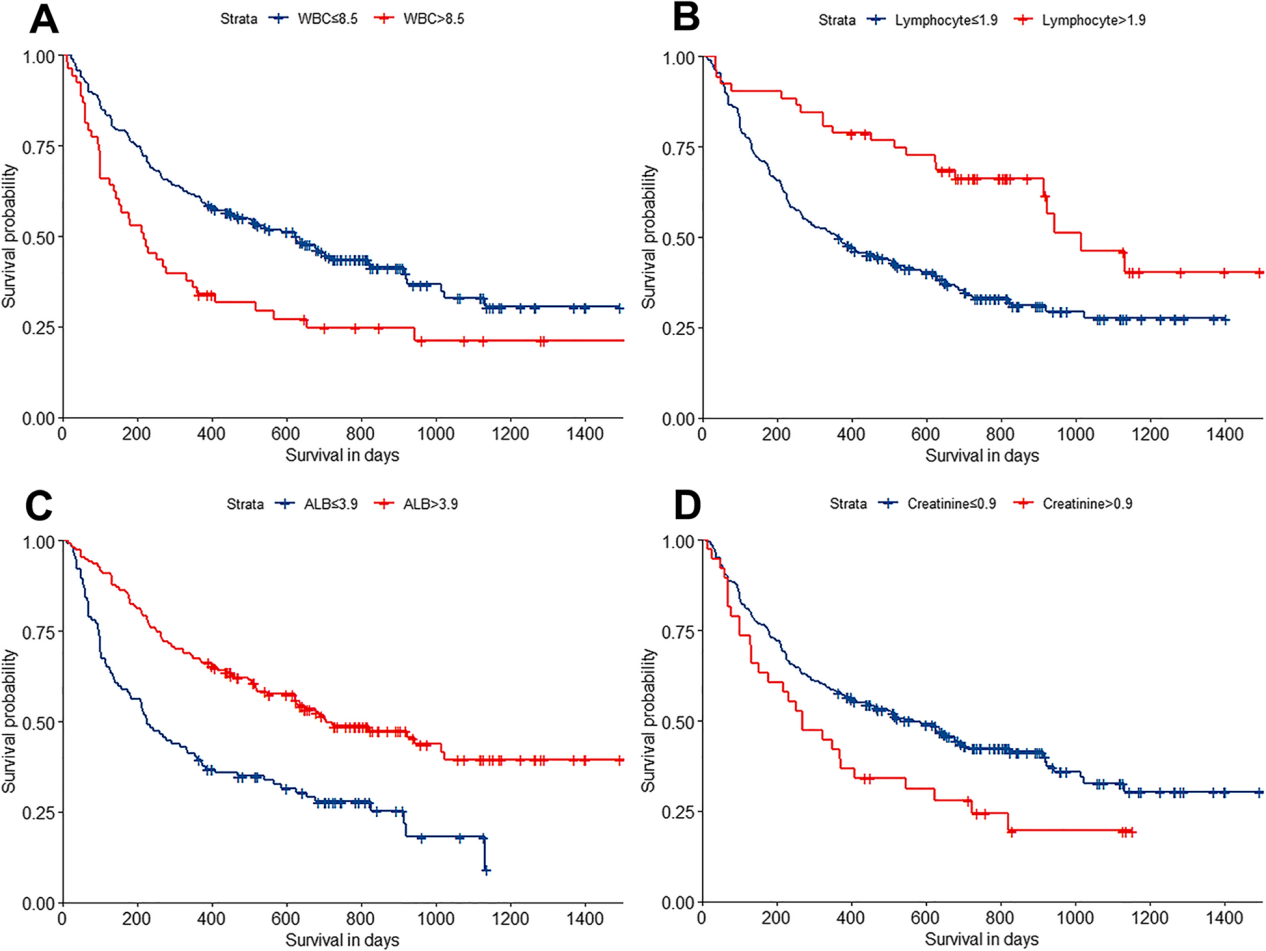
**a-d** Kaplan-Meier curves of overall survival based on four independent prognostic laboratory markers

According to the method described above [24], the optimal cutoff is 8.5 × 10^3^/μL for WBC, 1.9 × 10^3^/μL for lymphocyte count, 3.9 g/dL for albumin, and 0.9 mg/dL for creatinine. The survival curves were plotted using the cutoff values (Fig. 1).

### Construction and validation of the nomogram

**Fig. 2:**
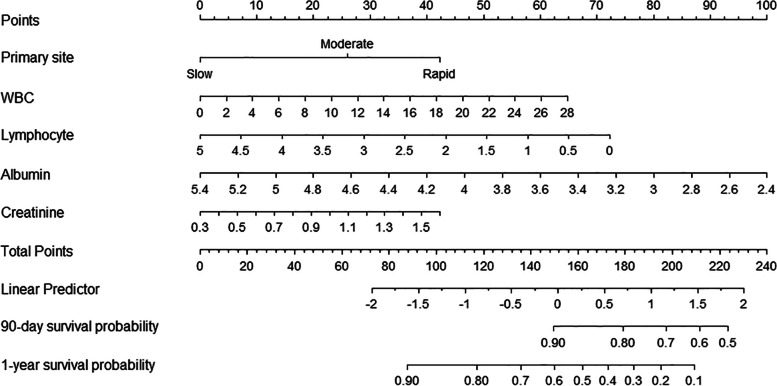
Our nomogram for survival prediction of patients with spinal metastases

According to the results of the multivariate Cox proportional hazards regression model, the five significantly independent prognostic factors, including the four predictive laboratory markers and primary tumor, were incorporated into the nomogram to predict the 90-day and 1-year survival probability. To obtain the corresponding point of each predictor, a straight line was drawn from each predictor upward to the points axis. The total point was calculated by summing up all the individual points. By directly drawing a line from the total point axis to the two axes on the bottom, the 90-day and 1-year survival probability after surgical treatment for spinal metastases is determined (Fig. 2).

**Fig. 3:**
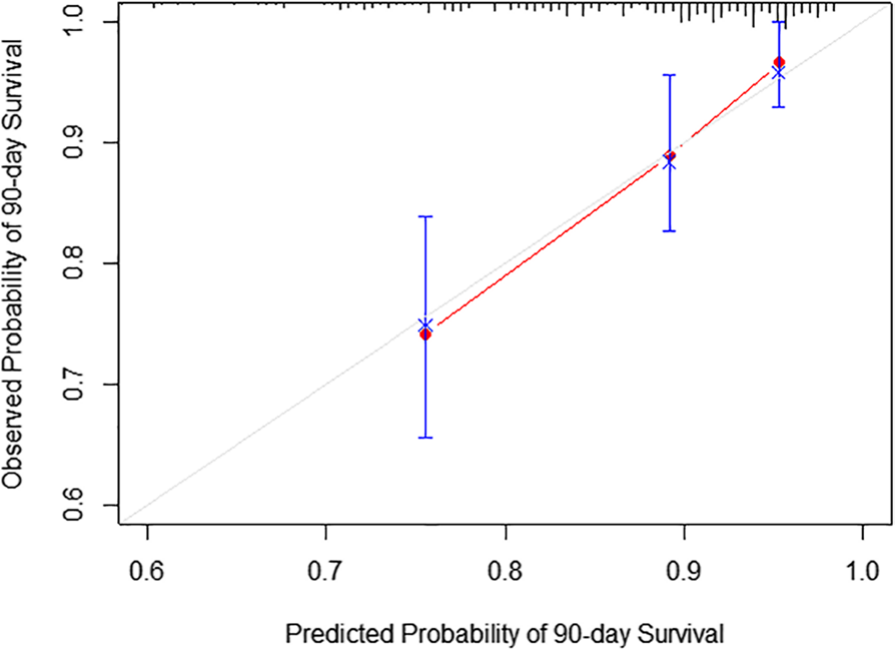
Calibration curve for 90-day survival prediction using our nomogram

**Fig. 4:**
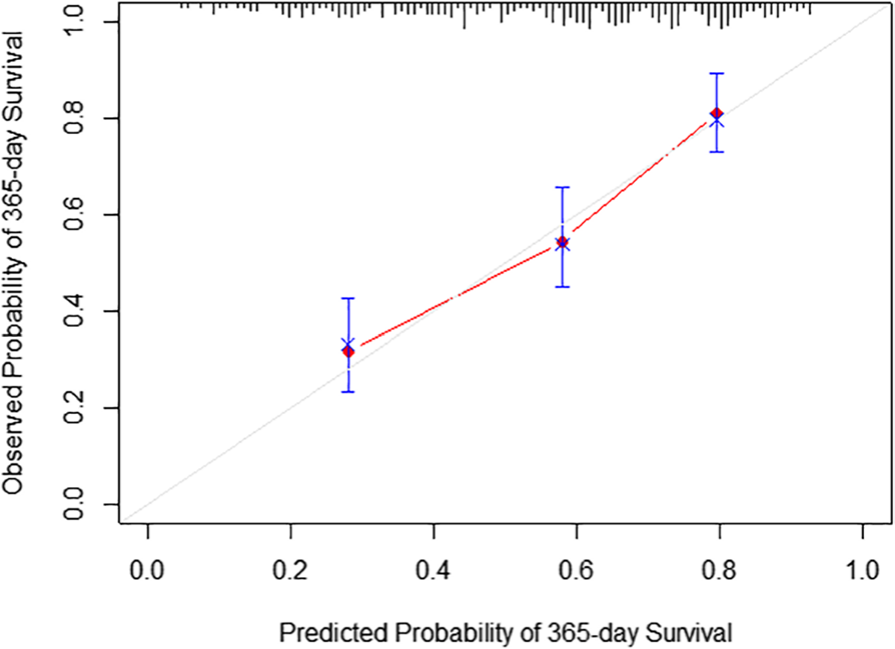
Calibration curve for 1-year survival prediction using our nomogram

The nomogram was validated with a C-index of 0.706 (95% CI, 0.702–0.710). The calibration curves showed favorable consistency between the predicted probability and the observed probability of the 90-day and 1-year overall survival (Fig. 3 and Fig. 4).

**Fig. 5:**
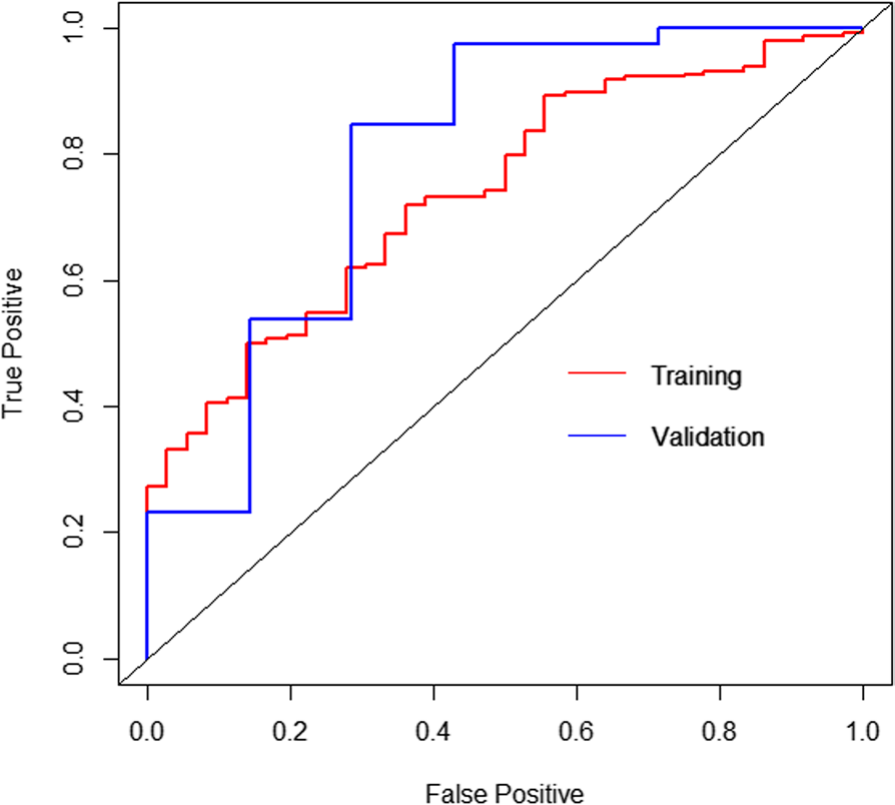
ROC curves of our nomogram at predicting 90-day survival in the training cohort and the validation cohort

**Fig. 6:**
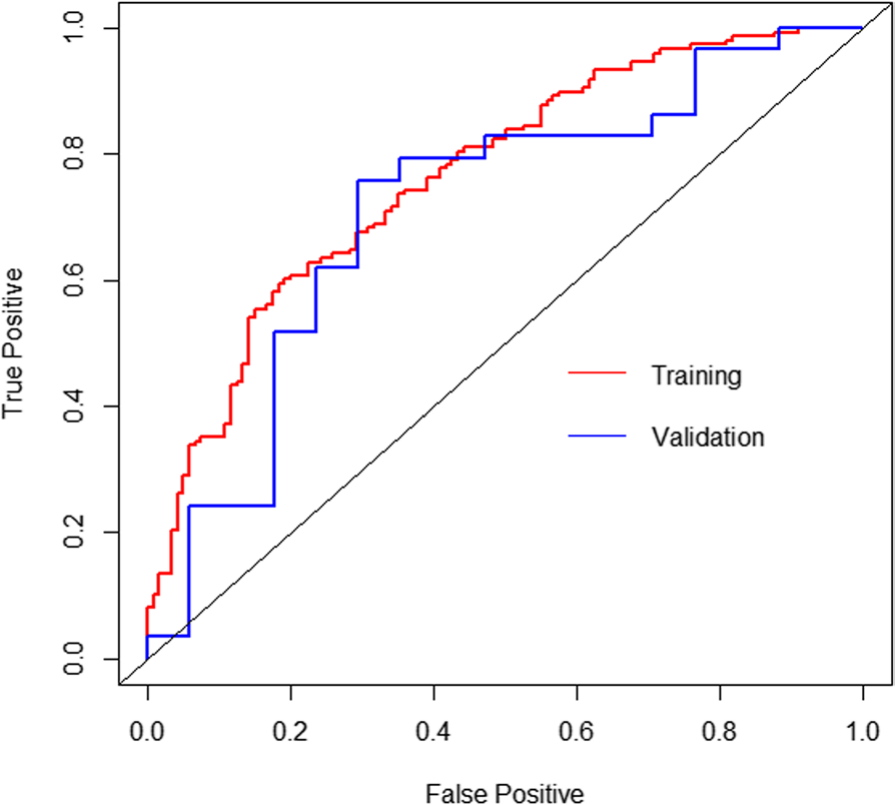
ROC curves of our nomogram at predicting 1-year survival in the training cohort and the validation cohort

For predicting 90-day survival after the index surgery in the present study, the AUC of our nomogram in the training group and the validation group was 0.740 (95% CI, 0.660–0.819) and 0.795 (95% CI, 0.568–1.000), respectively (Fig. 5). For predicting 1-year survival, the AUC of our nomogram in the training group and the validation group was 0.765 (95% CI, 0.709–0.822) and 0.712 (95% CI, 0.547–0.877), respectively (Fig. 6).

### Comparison with the existing scoring systems

**Fig. 7:**
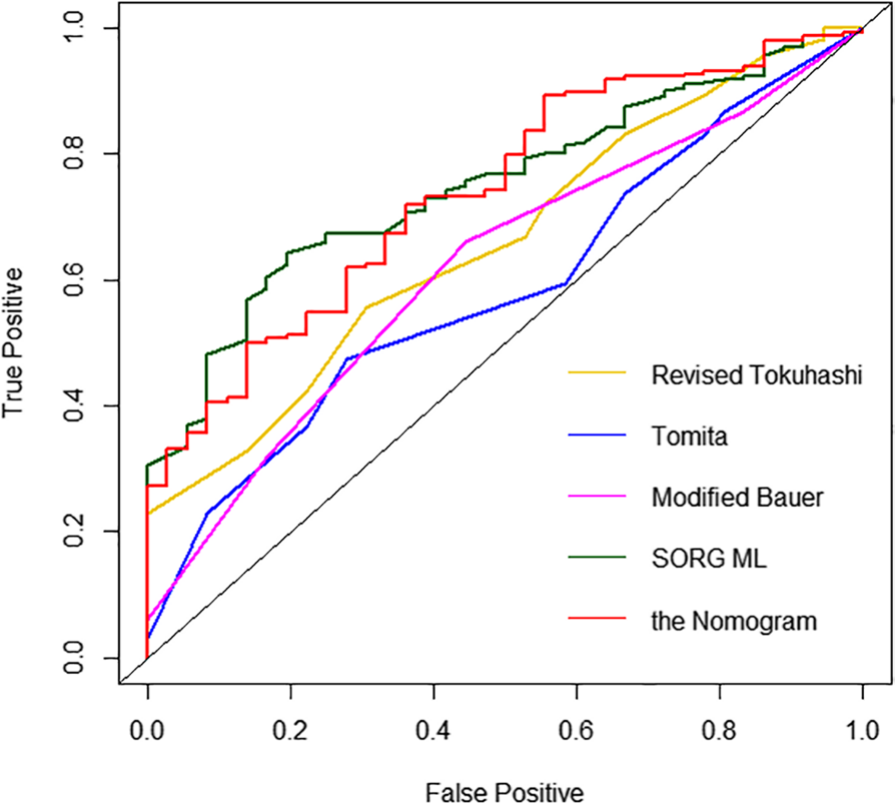
ROC curves of the revised Tokuhashi, Tomita, modified Bauer, SORG ML, and our nomogram at predicting 90-day survival

**Fig. 8:**
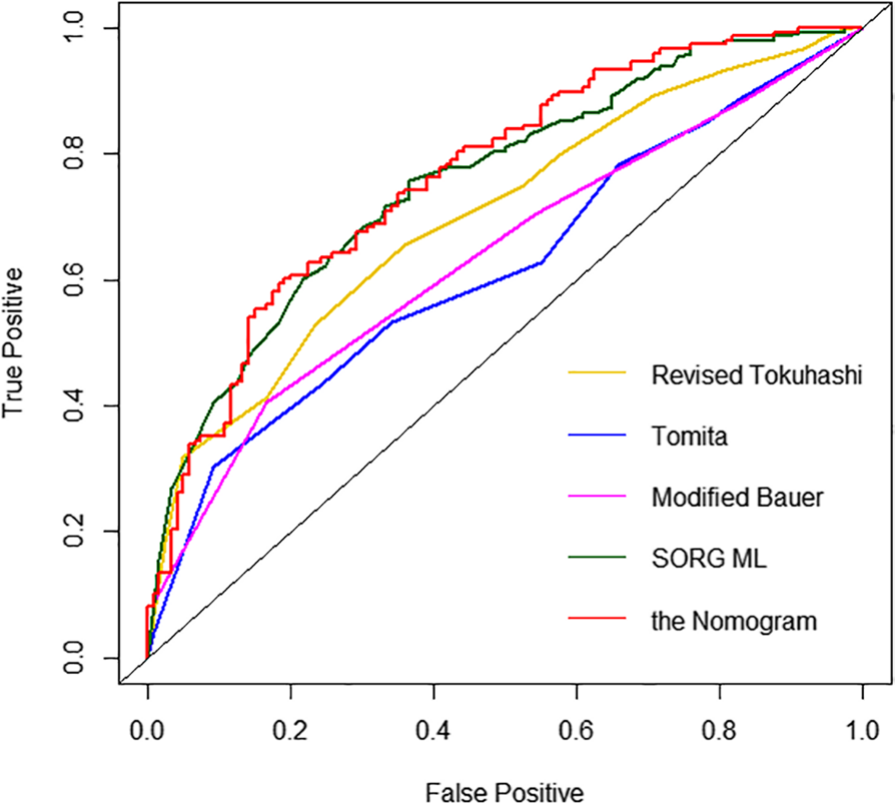
ROC curves of the revised Tokuhashi, Tomita, modified Bauer, SORG ML, and our nomogram at predicting 1-year survival

To compare the predictive ability of our nomogram with the existing scoring systems, the AUCs of Tomita, revised Tokuhashi, modified Bauer, and SORG ML were calculated using data from the training group. For predicting 90-day survival, the AUC of Tomita was 0.595 (95% CI, 0.494–0.687), the revised Tokuhashi was 0.650 (95% CI, 0.548–0.745), the modified Bauer was 0.618 (95% CI, 0.518–0.708), the SORG ML was 0.743 (95% CI, 0.666–0.817) (Table 4, Fig. 7). For predicting 1-year survival, the AUC of Tomita was 0.620 (95% CI, 0.552–0.689), the revised Tokuhashi was 0.702 (95% CI, 0.637–0.767), the modified Bauer was 0.646 (95% CI, 0.575–0.708), the SORG ML was 0.787 (95% CI, 0.730–0.838) (Table 4, Fig. 8). In the validation group, the prediction accuracy between our nomogram and other scoring systems was also performed (Table 5).

**Table 4:** Comparison of AUC for 90-day and 1-year survival prediction among different survival prognosis scores (the current nomogram, Tomita, revised Tokuhashi, modified Bauer, and SORG ML) in the development cohort

Scoring systems	All patients		Breast		Lung		Other	
	AUC	*P*	AUC	*P*	AUC	*P*	AUC	*P*
**AUC for 90-day survival prediction**								
the Nomogram	0.740 (0.660–0.819)	Ref	0.784 (NA)	Ref	0.625 (0.444–0.806)	Ref	0.725 (0.614–0.836)	Ref
Tomita	0.595 (0.494–0.687)	0.004	0.824 (NA)	0.107	0.465 (0.280–0.649)	0.006	0.578 (0.450–0.694)	0.033
Revised Tokuhashi	0.650 (0.548–0.745)	0.192	0.922 (NA)	NA	0.621 (0.437–0.805)	0.250	0.619 (0.510–0.728)	0.080
Modified Bauer	0.618 (0.518–0.708)	0.032	0.765 (NA)	NA	0.478 (0.323–0.633)	0.004	0.594 (0.473–0.716)	0.050
SORG ML	0.743 (0.666–0.817)	0.787	0.804 (NA)	NA	0.665 (0.504–0.801)	0.427	0.732 (0.635–0.828)	0.903
**AUC for 1-year survival prediction**								
the Nomogram	0.765 (0.709-0.822)	Ref	0.717 (0.551-0.882)	Ref	0.834 (0.738-0.930)	Ref	0.725 (0.644-0.806)	Ref
Tomita	0.620 (0.552-0.689)	< 0.001	0.566 (0.389-0.743)	0.024	0.413 (0.283-0.543)	< 0.001	0.653 (0.555-0.743)	0.020
Revised Tokuhashi	0.702 (0.637-0.767)	0.078	0.644 (0.449-0.839)	0.246	0.531 (0.396-0.667)	0.002	0.742 (0.657-0.813)	0.455
Modified Bauer	0.646 (0.575-0.708)	< 0.001	0.565 (0.392-0.737)	0.034	0.446 (0.325-0.567)	< 0.001	0.653 (0.552-0.744)	0.018
SORG ML	0.787 (0.730-0.838)	0.745	0.740 (0.583-0.886)	0.363	0.793 (0.679-0.879 )	0.973	0.759 (0.678-0.831)	0.584

*NA Not available to calculate the 95% confidence interval

**Table 5:** Comparison of AUC for 90-day and 1-year survival prediction among different survival prognosis scores (the current nomogram, Tomita, revised Tokuhashi, modified Bauer, and SORG ML) in the validation cohort

Scoring systems	All patients	
	AUC	*P*
**AUC for 90-day survival prediction**		
the Nomogram	0.795 (0.568–1.000)	Ref
Tomita	0.650 (0.533–0.767)	0.695
Revised Tokuhashi	0.518 (0.282–0.755)	0.076
Modified Bauer	0.626 (0.517–0.735)	0.799
SORG ML	0.709 (0.487–0.930)	0.477
**AUC for 1-year survival prediction**		
the Nomogram	0.712 (0.547–0.877)	Ref
Tomita	0.702 (0.550–0.856)	0.927
Revised Tokuhashi	0.515 (0.341–0.689)	0.118
Modified Bauer	0.653 (0.498–0.809)	0.527
SORG ML	0.767 (0.623–0.910)	0.454

## Discussions

Patients with spinal metastases experiencing mechanical instability, nerve compression, and pain can benefit from surgery [3, 25–27]. However, surgical decisions carry significant, non-negligible risk of surgical complications and treatment cost [6, 7, 9]. Accurate assessment of life expectancy is key to optimized treatments and objective physician-patient communication [8, 11, 13]. Effective identification and evaluation of prognostically predictive factor can greatly contribute to targeted patient care and avoiding overtreatment or undertreatment. The potential prognostic value of routinely collected laboratory markers has frequently been highlighted in many newer prediction algorithms due to improved accuracy, such as revised Katagiri [18], SORG nomogram [20], SORG ML [11], and New England Spinal Metastasis Score [19]. However, these algorithms use different laboratory items and subcomponent weights for analysis. Here, we systematically assessed potential laboratories markers and quantified their impact on prognosis for spinal metastases to address the discrepancies between such algorithms. Furthermore, we developed a nomogram based on significantly laboratory markers to reliably predict postoperative survival for patients with spinal metastases.

Serum albumin level is a well-established metric for assessing nutritional status, disease severity, and progression [28]. Hussain et al .[29] found that preoperative hypoalbuminemia was associated with increased risk of perioperative adverse events following surgical decompression of spinal metastases, including perioperative mortality, complication, transfusion, prolonged hospitalization, and non-home discharge. The NESMS [19], revised Katagiri [18], and SORG ML [11], have also incorporated serum albumin as a prognostic factor.

Anemia occurs frequently in cancer patients, especially in those undergoing chemotherapy and radiotherapy [30]. Cancer-induced anemia has a complex etiological nature [31]; studies have shown that it is a factor that adversely affects cancer patients’ survival [11, 32, 33]. Anemia can negatively impact survival by increasing patient frailty, complication risks, delaying initiation or failure of adjuvant therapy completion [31, 34]. Scoring systems incorporate hemoglobin level including the SORG classic [33], SORG nomogram [33], and SORG ML [11].

Elevated WBC indicates of systemic inflammation and approximately 1–10% of patients with nonhematopoietic malignancies develop tumor-related leukocytosis, which is associated with a poor prognosis [35, 36]. Inflammation in the tumor microenvironment facilitates cancer development and progression [16, 37]. Inflammatory conditions promote proliferation and survival of malignant cells, angiogenesis, and metastasis, subverting adaptive immune responses and altering hormone and chemotherapy efficacy [37]. Scoring systems incorporate WBC count including the SORG classic [33] and SORG nomogram [33]. Many studies have demonstrated that increased lymphocyte infiltration of tumors correlates with better response to cytotoxic therapy and better prognosis in cancer patients [16, 38]. NLR and PLR have been identified in previous publications as prognostic indicators [21]. In the present study, NLR and PLR were found to be significant in univariate analysis but not in multivariate analysis. Serum creatinine is a byproduct of muscle metabolism that is excreted by the kidneys. Elevated blood creatinine indicates active proteolytic activity and impaired renal function, serving as a poor prognostic factor for patients with malignancy [39, 40]. For spinal metastases, the SORG ML incorporated it into the algorithm [11].

To our surprise, our prediction model demonstrated satisfactory accuracy based only on laboratory markers and primary tumor histology, without other clinical predictors, such as visceral metastases, general condition, or neurological status. This a reasonable finding, since laboratory markers, body status, and tumor burden are closely linked [15, 16, 38, 39]. Furthermore, compared to other parameters, using laboratory markers for prognosis prediction is relatively objective, clear, and consistent [23]. The individual variations in patients with spinal metastases is large and some variables may be predictive for a specific subset of patients, but not others, reducing the overall reliability of the model.

Poor baseline general condition is considered as an adverse factor in many scoring systems [11, 19, 33, 41, 42]. However, it may be resolved in a relatively short period of time after the surgery, especially for patients with pathological fractures or mild neurological deficits [43]. Moreover, the intra- and interobserver variability may also affect the prediction accuracy [44]. Visceral metastasis is another important prognostic factor that has been used in the Tokuhashi [41, 45] and Tomita [46], classified into treatable and untreatable (by operation or trans arterial embolization). However, the relevant criteria are not clear, and a visceral metastasized lesion is rarely removed surgically for patients with spinal metastases. Classification of brain metastases as different from metastases from other organs is also controversial [13]. Due to differences in imaging tests for tumor staging, the results of systematic assessment can also vary over time and in different regions.

Compared to other existing prediction algorithms, our nomogram seems to have better prediction ability than Tomita, revised Tokuhashi, modified Bauer, and has comparable prediction ability compared to SORG ML. The accuracy of these scoring systems in the present study was similar to currently published literature [12, 13, 47]. Although this was a retrospective study, we believe that our finding are reliable. First, the results of laboratory data were less affected by the nature of the retrospective study. Second, patient follow-up was conducted prospectively by the hospital. Laboratory indicators are more objective and less influenced by examination methods when compared to predictors relying on imaging studys and subjective assessment by physicians. The prediction model built by data mining and combination of routinely collected laboratory markers will be more practical and more universal.

The findings of this study must be seen considering the following limitations. First, the study was based on a cohort from a single regional oncology center with risk of model overfitting, external validation is needed for the prediction model to prove the generalizability of the model. Second, we did not analyze some prognostic markers that are not routinely collected in clinical practice, such as C-reactive protein, and lactate dehydrogenase [18]. Prospective studies should include these tests in the protocol to expand the analytical power of the predictive model.

## Conclusion

Our study confirmed that many routinely collected laboratory markers can serve as promising predictive factors for postoperative outcomes of patients with spinal metastases. A nomogram based on primary tumor, WBC, lymphocyte count, albumin, and creatinine may accurately predict postoperative survival for patients with spinal metastases. In future work, whether active interventions guided by predictive laboratory markers will improve prognosis assessments for patients needs to be further investigated.

## Data Availability

The data that support the findings of this study are available on request from the corresponding author. The data are not publicly available due to privacy or ethical restrictions.

## Abbreviations

WBC: White blood cell
PLR: Platelet-to-lymphocyte ratio
NLR: Neutrophil-to-lymphocyte ratio
C-index: Concordance index
AUC: Area under curves
SORG ML: Skeletal Oncology Research Group machine-learning
ROC: Receiver operating characteristic
ECOG: Eastern Cooperative Oncology Group
NESMS: The New England Spinal Metastasis Score
